# Upper-limb kinematics and kinetics imbalances in the determinants of front-crawl swimming at maximal speed in young international level swimmers

**DOI:** 10.1038/s41598-020-68581-3

**Published:** 2020-07-15

**Authors:** Jorge E. Morais, Pedro Forte, Alan M. Nevill, Tiago M. Barbosa, Daniel A. Marinho

**Affiliations:** 1 Department of Sport Sciences, Instituto Politécnico de Bragança, Campus Sta. Apolónia, Apartado 1101, 5301-856 Bragança, Portugal; 2 Research Centre in Sports, Health and Human Development (CIDESD), University of Beira Interior, Covilhã, Portugal; 3 Higher Institute of Educational Sciences of the Douro, Penafiel, Portugal; 4 Faculty of Education, Health, and Wellbeing, University of Wolverhampton, Wolverhampton, UK; 5 University of Beira Interior, Covilhã, Portugal

**Keywords:** Bone quality and biomechanics, Anatomy

## Abstract

Short-distance swimmers may exhibit imbalances in their upper-limbs’ thrust (differences between the thrust produced by each upper-limb). At maximal speed, higher imbalances are related to poorer performances. Additionally, little is known about the relationship between thrust and swim speed, and whether hypothetical imbalances exist in the speed achieved while performing each upper-limb arm-pull. This could be a major issue at least while swimming at maximal speed. This study aimed to: (1) verify a hypothetical inter-upper limb difference in the determinants related to front-crawl at maximal swim speed, and; (2) identify the main predictors responsible for the swim speed achieved during each upper-limb arm-pull. Twenty-two male swimmers of a national junior swim team (15.92 ± 0.75 years) were recruited. A set of anthropometric, dry-land strength, thrust and speed variables were assessed. Anthropometrics identified a significant difference between dominant and non-dominant upper-limbs (except for the hand surface area). Dry-land strength presented non-significant difference (p < 0.05) between the dominant and non-dominant upper-limbs. Overall, thrust and speed variables revealed a significant difference (p < 0.05) between dominant and non-dominant upper-limbs over a 25 m time-trial in a short-course pool. Swimmers were not prone to maintaining the thrust and speed along the trial where a significant variation was noted (p < 0.05). Using multilevel regression, the speed achieved by each upper-limb identified a set of variables, with the peak speed being the strongest predictor (dominant: estimate = 0.522, p < 0.001; non-dominant: estimate = 0.756, p < 0.001). Overall, swimmers exhibit significant differences between upper-limbs determinants. The upper-limb noting a higher dry-land strength also presented a higher thrust, and consequently higher speed. Coaches should be aware that sprint swimmers produce significant differences in the speed achieved by each one of their upper-limbs arm-pull.

## Introduction

Researchers and practitioners are always keen to understand human locomotion in water^1^. Swimming is the most popular human locomotion in aquatic environments. In competitive swimming, the goal is to complete a given distance as quickly as possible. Within the several swimming strokes available, front-crawl raises most of the attention because it is the fastest, as well as, the one with more events included at major sports competitions^2,3^.

Swim speed is the net balance between drag and thrust forces acting on the swimmer’s body^4^. There is strong evidence on the effects of drag on human swimming^1,5^, whereas, the amount of evidence on thrust is far more limited. Theoretically there is a significant and positive relationship between thrust and swimmers’ level, i.e., larger thrust leads to faster speed^6^. Literature reports numerical studies (based on computational simulations) suggesting that swimmers achieve faster swim speeds whenever the thrust is increased^7^. However, little is known regarding such relationships using in-water experimental testing techniques during free swimming (i.e., simulating real swim conditions)^8^. In front-crawl, the upper-limbs arm-pulls account for 90% of the total propulsion^9^, and 88% of the total swim speed^10^. It is known that acceleration (necessary to obtain speed) depends on thrust, drag and mass. That is, speed depends on the thrust generated, hence thrust will be assumed to be a determining variable (independent) and speed as the dependent variable. As thrust and speed both contribute 88–90%^9,10^, it seems that the upper-limbs’ thrust is a strong determinant of swimming speed. Therefore, a lot of focus is given to the upper-limbs’ kinematics and kinetics, since a larger thrust production is positively associated to a faster swim^8^.

At maximal speeds, it could be expected that short-distance swimmers (in both short- and long-course swimming pools) should produce similar amount of thrust by both upper-limbs. These swim events take a short amount of time^11^. Thus, one can argue that swimmers are able to keep a steady thrust output by both upper-limbs. Notwithstanding, it was reported that the best performances in front-crawl were related to smaller imbalances between both upper-limbs^12^. However, studies reported mixed findings at least in tethered thrust force. Morouço et al.^13^ showed non-significant differences between upper-limbs’ in-water thrust. In contrast, dos Santos et al.^12^ reported significant imbalances between upper-limbs’ thrust. Moreover, it was argued that tethered swimming may not be an accurate method to assess upper-limbs’ imbalance^14^. As the swimmer is tethered at hips, this may not accurately represent the effect of the thrust on the acceleration of the swimmer’s center of mass^15^. Moreover, the absent of displacement could induce changes in the swimmer’s stroke pattern, creating mechanical constraints^16^. Hence, based on such mixed findings and limitations of tethered testing due to its questionable outputs, one can deduce that this topic remains unclear.

It is a fairly standard procedure to assess swim speed as a mean value during a given distance^2^. I.e., it is assumed that during an all-out short trial of for instance 25–50 m length swimmers do not significantly change their swim speed. Swimmers would be able to keep a steady maximal power output over the trial and therefore a uniform speed^17^. Literature reports variations in speed from lap to lap in short distance events such as the 100 m freestyle^18^. It was noted that there is a significant variation in speed and swim pace between laps^3^. However, there is no evidence on the variability of speed and it’s determinant factors within each lap.

Additionally, it is unclear if at all-out pace, swimmers keep up displacing at steady speed while producing thrust by dominant and non-dominant arm-pulls^19^. As mentioned earlier, literature reports mixed findings on upper-limbs thrust^12,13^. That is, at all-out speed, swimmers may (or may not) exhibit imbalances in their thrust. Nevertheless, there is no evidence whether swimmers achieve similar swim speeds producing thrust by dominant and non-dominant arm-pulls. In addition, there is no insight regarding the relationship between the thrust produced by each upper-limb and the swim speed achieved while performing the correspondent arm-pull.

Therefore, the main aims of this study were to: (1) verify whether a hypothetical inter-upper limb difference in the determinant factors (including anthropometrics, dry-land strength, kinematics, and thrust) might be related to front-crawl at maximal swim speed, and; (2) identify the main predictors responsible for the swim speed achieved during each upper-limb arm-pull. It was hypothesized that: (1) a non-significant inter-upper limb effect would be found for the swim speed and determinant factors, and; (2) that swim speed predictors would be based on an interaction of several determinant factors.

## Results

### Inter-limb differences

Table 1 presents the comparison between the dominant and non-dominant upper-limb in each variable assessed. Anthropometrics reveal a significant difference with a small effect size between upper-limbs (except for the hand surface area—HSA). Non-significant difference with a small effect size was found between the dominant and non-dominant upper-limbs in dry-land strength. As for the in-water variables, the mean swim speed (v_mean_) revealed the highest inter-limb difference (t = 4.69, p < 0.001, d = 0.44) (Table 1).

**Table 1:** Descriptive statistics (mean ± one standard deviation, SD) for the anthropometric, dry-land strength, and in-water variables.

	Mean ± 1SD		Inter-limb difference		Dominant		Non-dominant	
	Dominant	Non-dominant	t-test (p)	d	F-ratio (p)	η^2^	F-ratio (p)	η^2^
Arm (cm)	31.93 ± 2.85	31.61 ± 2.89	2.38 (0.027)	0.11				
Forearm (cm)	27.91 ± 1.96	27.55 ± 1.99	2.09 (0.049)	0.19				
HSA (cm^2^)	139.04 ± 10.09	140.57 ± 11.72	− 1.65 (0.115)	0.14				
Handgrip (kg)	45.63 ± 3.46	44.88 ± 6.56	0.76 (0.454)	0.07				
v_mean_ (m s^−1^)	1.62 ± 0.08	1.58 ± 0.10	4.69 (< 0.001)	0.44	9.11 (0.001)	0.11	4.67 (0.016)	0.05
v_peak_ (m s^−1^)	1.86 ± 0.13	1.78 ± 0.11	4.27 (< 0.001)	0.66	4.01 (0.036)	0.09	2.95 (0.066)	0.07
dv (%)	9.82 ± 3.81	9.06 ± 4.00	1.74 (0.087)	0.20	1.40 (0.259)	0.02	1.09 (0.345)	0.01
UST (s)	0.81 ± 0.10	0.81 ± 0.08	0.02 (0.986)	0.00	8.40 (0.002)	0.02	1.55 (0.227)	0.01
F_mean_ (N)	40.21 ± 5.75	38.48 ± 5.99	2.03 (0.046)	0.31	8.83 (0.001)	0.05	0.84 (0.404)	0.01
F_peak_ (N)	63.21 ± 9.79	64.29 ± 8.89	− 0.85 (0.400)	0.11	1.43 (0.252)	0.01	0.60 (0.549)	0.01
dF (%)	41.91 ± 9.13	47.83 ± 9.91	− 4.02 (< 0.001)	0.64	2.62 (0.105)	0.04	1.22 (0.305)	0.02

It is also presented the inter-limb difference between dominant and non-dominant variables, and the variation (repeated measures) of the in-water variables during three consecutive cycles.

*p* significance value, *d* Cohens d (effect size index), *η*^*2*^ eta square effect size, *HSA* hand surface area, *v*_*mean*_ mean swim speed, *v*_*peak*_ peak swim speed, *dv* intra-cyclic variation of the swim speed, *UST* underwater stroke time, *F*_*mean*_ mean thrust of the arm-pull, *F*_*peak*_ peak thrust, *dF* intra-cyclic variation of the thrust, (−) negative symbol in the t-test indicates that dominant side is lower than the non-dominant.

### Swim kinetics and kinematics variation

**Figure 1:**
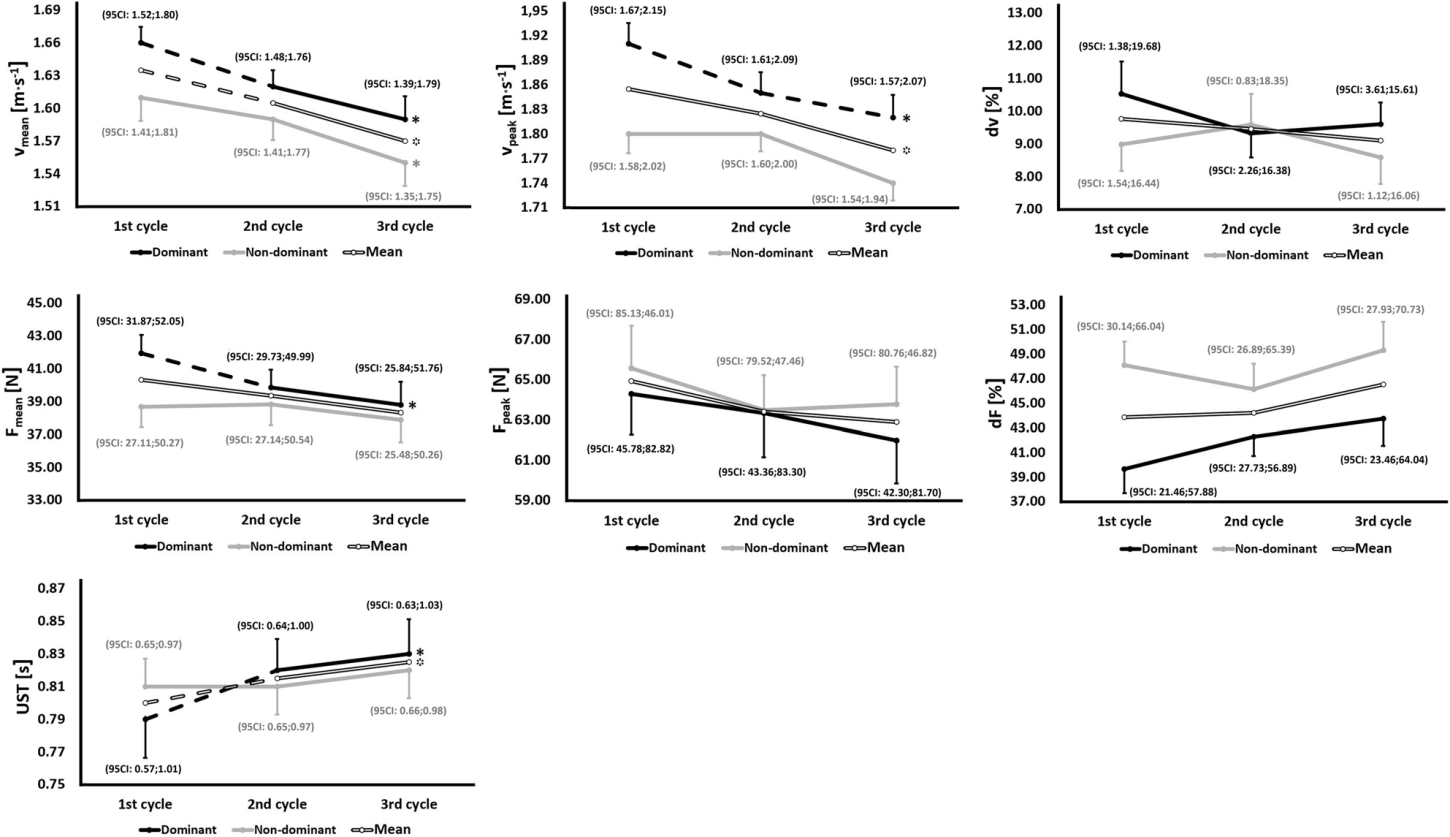
Variation of the variables assessed during three consecutive stroke cycles. *v*_*mean*_ swim speed, *v*_*peak*_ swim speed peak, *dv* intra-cyclic variation of the swim speed, *F*_*mean*_ mean thrust of the arm-pull, *F*_*peak*_ peak thrust, *dF* intra-cyclic variation of the thrust, *UST* underwater stroke time. Bars—standard error; Solid lines—non-significant differences between consecutive cycles; Dash lines—significant differences between consecutive cycles (p < 0.05); *Differences between the first and third cycle (p < 0.05); in each stroke cycle is presented the 95% confidence interval (95 CI).

The variation of the in-water variables (for each upper-limb) is depicted in Fig. 1 and Table 1. The highest and significant variation between the three cycles was found in the v_mean_ for both dominant and non-dominant upper-limbs (dominant: F = 9.11, p = 0.001, η^2^ = 0.11; non-dominant: F = 4.67, p = 0.016, η^2^ = 0.05) (Table 1). The v_mean_ achieved by the dominant upper-limb presented a significant difference (p < 0.05) between the first and the second stroke cycle, and between the first and the third. As for the v_mean_ achieved by the non-dominant upper-limb a significant difference (p < 0.05) was verified only between the first and the third stroke cycle (Fig. 1). Moreover, it was verified a trend to a v_mean_ and peak swim speed (v_peak_) decrease in both upper-limbs (Fig. 1).

The thrust of the dominant upper-limb presented a similar profile as the v_mean_ (Table 1 and Fig. 1). However, the thrust of the non-dominant limb presented a non-significant variation (F = 0.84, p = 0.404, η^2^ = 0.01) (Table 1 and Fig. 1). Nevertheless, we observed a decrease in both dominant and non-dominant upper-limb thrust. The underwater stroke time (UST) presented a significant variation for the dominant upper-limb (F = 8.40, p = 0.002, η^2^ = 0.02), but not for the non-dominant. A trend to increase the amount of time performing the in-water phase was observed for both upper-limbs (being more pronounced in the dominant upper-limb) (Fig. 1).

### Swim speed determinants

The results of the multilevel regression analysis predicting swimming speeds achieved by the dominant, and non-dominant upper-limb are reported in Table 2. In both models, the v_peak_ showed the highest contribution (dominant: estimate = 0.522, 95 CI 0.404; 0.640; non-dominant: estimate = 0.756, 95 CI 0.678; 0.834) (Table 2). These slope parameters suggest that an increase in one unit (m s^−1^) in the v_peak_ would led to an increase in v_mean_ of 0.522 and 0.756 m s^−1^ for the dominant and non-dominant upper-limb, respectively.

**Table 2:** Fixed effects of the final model computed with standard errors (SE), 95% confidence intervals (95 CI), test-score (t-score), and significance value (p).

Fixed effect	Estimate (SE)	95 CI	t-score	p value
**Dominant swim speed**				
UST	− 0.241 (0.071)	− 0.380; − 0.102	3.394	< 0.001
Handgrip	0.005 (0.002)	0.001; 0.009	− 2.500	< 0.001
v_peak_	0.522 (0.060)	0.404; 0.640	− 8.700	< 0.001
dv	− 0.013 (0.002)	− 0.017; − 0.010	6.500	< 0.001
**Non-dominant swim speed**				
Decimal age	− 0.049 (0.013)	− 0.074; − 0.024	3.769	< 0.001
Arm	0.009 (0.004)	0.001; 0.017	− 2.250	0.018
HSA	− 0.003 (0.001)	− 0.005; − 0.001	3.000	< 0.001
F_mean_	0.002 (0.001)	0.00004; 0.00396	− 2.000	0.029
v_peak_	0.756 (0.040)	0.678; 0.834	− 18.900	< 0.001
dv	− 0.013 (0.001)	− 0.015; − 0.011	13.000	< 0.001

*UST* underwater stroke time, *v*_*peak*_ peak swim speed, *dv* intra-cyclic variation of the swim speed, *HSA* hand surface area, *F*_*mean*_ mean thrust of the arm-pull.

## Discussion

The aim of this study was to explore whether a hypothetical upper-limb difference exists in anthropometric, kinematic, and thrust determinants related to front-crawl swim at maximal swim speed, and to identify the main predictors responsible for the swim speed achieved during each upper-limb arm pull. Main findings showed a significant difference for the anthropometrics, thrust, and speed achieved by each upper-limb. Swimmers also tend to decrease their thrust and speed achieved by each upper-limb along the swim bout (both upper-limbs and average). The v_mean_ achieved by each upper-limb (and the average) was determined by an interaction of several determinant factors.

Upper-limbs are responsible for the major production of thrust and speed^9,10^. Therefore, one can argue that a meaningful difference between sides in anthropometrics and dry-land strength could lead to differences in the performance (thrust and speed) achieved by each upper-limb. Overall, a significant difference between sides was verified for the anthropometric variables, but not for dry-land strength. Nevertheless, all variables presented higher values in the dominant side, except the HSA. Curiously, this hand asymmetry related to limb dominance is reported in the literature for the general population^20^, and also within swimmers^14^. Regarding the handgrip (dry-land strength), the dominant side also presented higher values (but not significant). A study that assessed hand force asymmetries in a similar age-group revealed mixed findings, where a part of the sample presented a non-significant side effect, and others a significant side effect^21^. Since these asymmetries could be responsible for inducing musculoskeletal strength imbalances, it seems that coaches and swimmers should be aware and advised for this issue.

Significant differences were noted in F_mean_ and the speed achieved by each upper-limb (v_mean_ and v_peak_). The dominant upper-limb achieved a higher F_mean_ in comparison to the non-dominant upper-limb over the three back-to-back cycles. This higher F_mean_ might be related to dry-land strength^21^. Swimmers did present higher dry-land strength values by the dominant upper-limb in comparison to the non-dominant. Interestingly, the non-dominant upper-limb delivered a higher (but not significant) F_peak_ during the tree cycles. Even though there is no evidence, one can speculate that this can be related to a lower propelling efficiency by the non-dominant limb. As far as our understanding goes there is not an assessment of propelling efficiency variations by the dominant and non-dominant limbs. However, if it is accurate that the non-dominant limb might be less efficient, then swimmers could try to outperform the thrust being produced as a trade-off motor control strategy.

Regarding the speed achieved while performing each arm-pull, a significant difference for both v_mean_ and v_peak_ was noted. The dominant upper-limb did present a higher v_mean_ showing a similar profile to the F_mean_ (i.e., higher F_mean_ imposed a higher v). Indeed, evidence in the literature notes that higher thrust is positively associated to a higher v_mean_^22^. On the other hand, the dominant limb revealed the highest v_peak_ but a lower F_peak_ in comparison to the non-dominant. Consequently, the non-dominant limb achieved a higher F_peak_ but a lower v_peak_. Overall, the trend suggests that whenever a large thrust is produced, a corresponding high swim speed is noted. However, resistive forces acting on the swimmer’s body also play a determinant role during the arm-pull^23^. Swimmers may not present similar alignments or motor dominance when performing both arm-pulls which may lead to different resistive forces and hence different speed while performing both arm-pulls^15^. Indeed, when one side of the body produce thrust the other side should be responsible for maintaining a good alignment decreasing the fontal surface area, and hence minimizing the speed decrease^19^.

Overall, a significant time effect was verified for the thrust and speed variables, where a decrease was verified. When a swimmer is performing at maximum power, it could be assumed that the swimmer is able to maintain the mean thrust and speed over a 25 m trial or lap. However, it seems that swimmers are prone to decrease thrust and speed over the trial^12^. This might be related to the swimmer’s energetic profile. Previous studies have shown that swim sprinters tend to increase the energy cost between laps^18^. However, present data showed that even within the same lap or trial swimmers are not capable of maintaining a steady swim speed, which can be related to the incapacity of producing a constant mechanical power.

The swim speed achieved by the dominant upper-limb was determined by an interaction of dry-land strength and in-water kinematic variables. Dry-land strength has a positive and significant effect on swimming performance^24^. However, this assumption was shown for the full stroke cycle not relating to each upper-limb. Our data indicated that the dominant upper-limb achieved the highest dry-land strength, and this allowed the swimmers to present a lower UST (also retained as a determinant) and consequently higher thrust. Literature showed that shorter UST is related to a higher amount of thrust^25^. Therefore, swimmers that present higher dry-land strength are prone to produce larger amounts of thrust, leading to faster speeds. Mean swim speed was also determined by v_peak_ (positive effect) and dv (negative effect). Theoretically, achieving a high swim speed could lead to a steady mean swim speed^26^. However, the mean speed is based on the balance between maximal and minimal speeds (i.e., dv). Indeed, it was noted that an increase in the dv had a negative effect on speed^27^. Therefore, swimmers should be advised to reach high in-water accelerations during the entire arm-pull (i.e., thrust) in order to minimize the lowest speed achieved, and hence a low dv.

The non-dominant upper-limb retained the same kinematic variables as the dominant one (i.e., v_peak_ and dv). Hence, the rationale is similar to the dominant upper-limb. Additionally, the F_mean_ was also retained as a determinant. If the dominant upper-limb presented an indirect relationship between thrust and speed (being mediated by the UST), the speed achieved by the non-dominant upper-limb was determined by thrust. Therefore, it can be highlighted that indeed higher thrust imposes faster swim speeds. Anthropometric variables also determined the speed achieved by the non-dominant upper-limb (namely the arm and HSA). Literature reports that greater body dimensions lead to higher swim speed^14^. Interestingly, the HSA had a negative effect on the swim speed achieved by the non-dominant upper-limb. However, one can suggest that at this performance level (national and international level swimmers), anthropometrics (i.e., HSA) is not the most important factor compared with younger counterparts^5^. Nonetheless, the hand orientation and its pitching and sweepback angles may be considered more important^28^. Decimal age also presented a negative effect, i.e., speed achieved by this upper-limb decreased with an age increase. It seems that swimmers subconsciously assume that their main producer of speed is their dominant upper-limb based in the fact that their dominant side is stronger (dry-land strength). This seems to be a major issue regarding maximal swim speeds, where asymmetries in the speed achieved by each upper-limb are expected to be minimized during time^15^. In this sense, coaches should be advised that swimmers may present such handicaps, especially in maximal speeds.

The upper-limb that achieved a higher dry-land strength also achieved a higher thrust. Thus, it seems that a positive relationship exists between these two factors. Altogether, whenever asymmetries in thrust, and consequently in speed between upper-limbs are verified, this reinforces the need for coaches and swimmers to develop training programs (in-water and dry-land) dedicated to reduce such imbalance. Indeed, literature reported that smaller asymmetries were related to best performances^12^. Coaches should also be advised that during a swim trial or lap, swimmers are not capable of maintaining similar indexes of thrust and speed, and the v_mean_ achieved by each upper-limb rely on different determinants.

The present thrust data was collected based in a pressure sensor system placed in the swimmer’s hands (please see the “Methods” section—“Thrust”). This system allows researchers to measure directly the thrust applied by the swimmer’s hand in the water, and hence gives an instantaneous output of the force generated to promote displacement. Moreover, our outputs are in accordance with the ones showed by numerical simulations^28^. In contrast, the tethered method presents constrictions mentioned earlier (please see the introduction section): (1) as the swimmers promote thrust with their hands, the measurement performed in the hips may not accurately represent the thrust generated^15^, and; (2) stroke pattern constraints^16^. Based on these assumptions, it can be indicated that this experimental approach (pressure sensor) measures thrust with higher accuracy than the tethered method. Nonetheless, the tethered method can be useful to indicate whether there are differences between the force generated by each upper-limb. It might be considered as a limitation of this research that: (1) outputs only refer to maximal speed and are limited to this specific age-group, and; (2) the selection of the handgrip test to measure the upper-limbs’ dry-land strength, instead of an isokinetic dynamometer. Future research designs might consider measuring the total arm-pull phase (in-water time), and also break it down into its sub-phases (downsweep, insweep, and upsweep) to have a deeper insight.

Swimmers presented significant differences in their anthropometrics, thrust, and speed achieved by the upper-limbs. Non-significant differences were noted in dry-land strength. Swimmers were not able to maintain their thrust and speed performances during the trial in both upper-limbs. The speed achieved by each upper-limb was determined by an interaction of key-factors, notably related to thrust and kinematics. Researchers and practitioners should be aware that sprint swimmers achieve significant differences in the speed recorded by their dominant and non-dominant upper-limbs’ arm-pull. We suggest that such differences may hinder an eventual improvement in speed.

## Methods

### Participants

The sample was composed of 22 male swimmers (demographics: 15.92 ± 0.75 years; 68.93 ± 6.99 kg of body mass; 1.77 ± 0.06 m of height; 1.83 ± 0.08 m of arm span; 566.77 ± 56.83 FINA points in the short course meter 100 m freestyle event). The swimmers were recruited from a national team at the end of the second macrocycle (peak performance), and had more than 5 years of competitive experience and performed six to seven training sessions per week, plus at least one dry-land strength and conditioning session per week. Inclusion criteria were as follows: (1) front-crawl sprint specialists, and; (2) present an opposition front-crawl arm coordination (please report to the design section). Parents or guardians, and the swimmers themselves signed an informed consent form. All procedures were in accordance to the Declaration of Helsinki regarding human research. The University of Beira Interior Ethics Board also approved the research.

### Design

This was a cross-sectional study. The swimmers’ hand dominancy was assessed by self-report as suggested elsewhere^14^. Before the testing, the athletes underwent familiarization sessions as part of their in-water and strength and conditioning measurements. The dry-land strength testing was performed by a certified strength and conditioning coach. Prior to the in-water data collection, swimmers performed a standardized 1,000 m warm-up. All in-water data was collected during three consecutive stroke cycles between the 11th and 24th m. Swimmers were instructed to hold their breath during such intermediate distance in order to avoid modifications in coordination due to breathing. To ensure a proper analysis of each stroke (i.e., dominant and non-dominant) and without any kind of bias (i.e., initiation of the non-dominant stroke while performing the dominant one: super-position inter-limb coordination, where an influence of an upper-limb speed would affect the other one and vice-versa), only swimmers presenting an opposition arm coordination were included in the analysis as mentioned earlier. This arm coordination occurs when one hand enters the water at the same time that the opposite hand exits the water^29^. The in-water experimental testing took place in a 25 m indoor swimming pool (water temperature: 27.5 °C; air temperature: 26.0 °C; relative humidity: 66%).

### Anthropometrics

Each upper-limb length and hand surface area (HSA) were measured by digital photogrammetry. For the upper-limbs’ length light markers were placed on the acromion, lateral epicondyle, and styloid process. Afterwards the arm (in cm) was measured between the acromion and the lateral epicondyle, and the forearm (in cm) between the lateral epicondyle and the styloid process (ICC = 0.990)^14^. For the HAS (in cm^2^), swimmers placed each hand on the scan surface of a copy machine. A 2D calibration pole was also placed on the scanning surface. The scan file was then exported to a laptop. The distances and surface areas were measured with a dedicated software (Universal Desktop Ruler, v3.8, AVPSoft, USA) (ICC = 0.989)^30^.

### Dry-land strength

The isometric handgrip test was chosen as the dry-land strength variable^31^. This presents important advantages in its application in youth swimmers: (1) non-invasive measurement; (2) allows the researcher to measure the upper-limbs’ strength separately (i.e., dominant vs non-dominant); (3) is highly correlated with strength and power in other muscular groups from the same sagittal side, and; (4) it’s simple and easy to apply in training contexts^32^. A digital hand dynamometer (Lafayette Instrument, 5030D1, USA) was used to measure the isometric handgrip (in kg) of dominant and non-dominant limbs. Swimmers were instructed stay in the orthostatic position, with both upper-limbs in extension along the trunk. Afterwards, they were asked to produce their maximal grip with the upper-limb in extension. Three trials (ICC = 0.989) were performed with a two 2 min rest in-between each trial^32^. The best score was used for further analysis.

### Stroke kinematics

**Figure 2:**
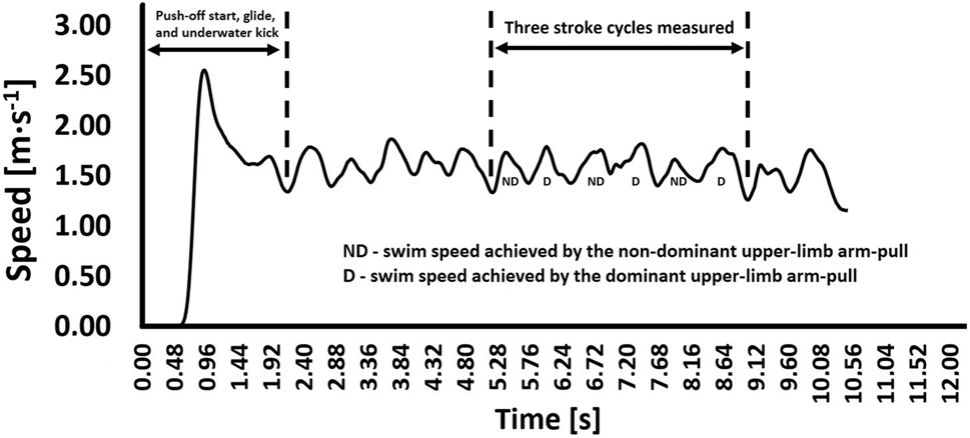
Example of a swimmer time-speed curve indicating the arm-pull (i.e., in-water phase) of each upper-limb (i.e., dominant and non-dominant). Whenever an upper-limb is performing the arm-pull the other is performing the recovery phase (opposition arm coordination).

Swimmers were instructed to perform three all-out trials at front-crawl with a push-off start. A mechanical apparatus (Swim speedo-meter, Swimsportec, Hildesheim, Germany) was attached to the swimmers’ hip^5^. An in-house built software (LabVIEW, v. 2010) was used to acquire (*f* = 50 Hz) and display speed-time data over each trial. Data was exported from the speedo-meter to interface by a 12-bit resolution acquisition card (USB-6008, National Instruments, Austin, Texas, USA). Then, it was imported into a signal processing software (AcqKnowledge v. 3.9.0, Biopac Systems, Santa Barbara, USA). A video camera, (Sony FDR-X3000, Japan) synchronized to the speed-time data, filmed the swimmers in the sagittal plane to identify the stroke cycle phases (i.e., arm-pull—time between the hand’s entry in the water and exit; recovery phase—time between the hand’s exit and water entry). Figure 2 depicts an example of a swimmer’s time-speed curve.

For each stroke (i.e., dominant and non-dominant), the mean swim speed (v_mean_, in m s^−1^), peak swim speed (v_peak_, in m s^−1^), the intra-cyclic variation of the horizontal swim speed (dv, in %), and the underwater stroke time (UST, in s) were assessed. The v (m s^−1^) was retrieved from the software in each specific point. The v_peak_ was considered the highest swim-speed value achieved during each stroke. The dv was computed as:

1 $$ dv = \frac{{\sqrt {\frac{{\mathop \sum \nolimits_{i} \left( {v_{i} - \overline{v}} \right) \cdot F_{i} }}{n}} }}{{\frac{{\mathop \sum \nolimits_{i} v_{i} \cdot F_{i} }}{n}}} \times 100 $$

where dv is the intra-cyclic variation of the horizontal swim speed (%), v is the mean swimming speed (m s^−1^), v_i_ is the instant swimming speed (m s^−1^), F_i_ is the acquisition frequency, and n is the number of observations^5^. The UST was computed as the time spent between the hand’s entry in the water and its exit.

### Thrust

**Figure 3:**
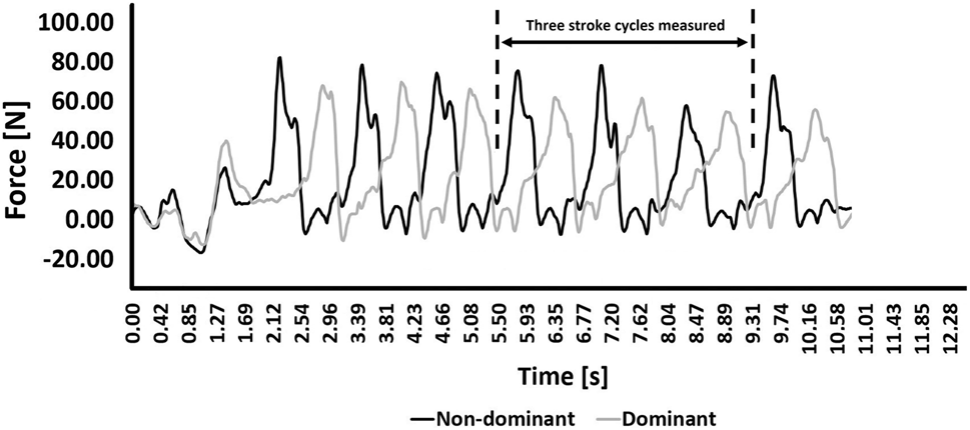
Example of a swimmer time-force curve indicating the force (i.e., thrust) that each upper-limb produced during the trial.

The thrust was assessed concurrently to kinematics (the same three maximal all-out trials of 25 m at front crawl with a push-off). A force data acquisition equipment (Aquanex + Video, Swimming Technology Research, USA) was used to measure thrust (*f* = 100 Hz). Such sensors were placed between the third and fourth metacarpals to measure the pressure differential between the palmar and dorsal surfaces. At the beginning of each trial, swimmers were asked to keep their hands immersed at the waistline for 10 s in order to calibrate the system with the hydrostatic pressure values. The underwater camera was placed on the wall of the swimming pool, recording the participants in the transverse plane. The sensors were connected to an A/D converter connected to a laptop on the pool floor with the Aquanex software (Aquanex v. 4.2 C1211, Richmond, USA)^14^. Afterwards, time-force series were imported into a signal-processing software (AcqKnowledge v. 3.9.0, Biopac Systems, Santa Barbara, USA). Signal was handled with Butterworth fourth order low-pass filter (cut-off: 5 Hz). Figure 3 depicts an example of a swimmer’s time-force curve. For each dominant and non-dominant arm-pull, the mean propulsive force (F_mean_, in N), and the peak force (F_peak_, in N), were analyzed. Afterwards, the intra-cyclic force variation (dF, in %) was computed based on Eq. (1).

### Statistical analysis

The Shapiro–Wilk and the Levene tests were used to assess the normality and homocedasticity, respectively. The mean plus one standard error were computed as descriptive statistics for all variables (arm, forearm, HSA, handgrip, v_mean_, v_peak_, dv, UST, F_mean_, F_peak_, dF).

Paired sample t-test (p < 0.05) was used to verify the presence of a side effect (i.e., difference between the dominant and non-dominant limbs) for all variables computed. Cohen’s d was selected as standardized effect size, and interpreted as: (1) small effect size 0 ≤|d|≤ 0.2; (2) medium effect size if 0.2 <|d|≤ 0.5 and; (3) large effect size if |d|> 0.5^33^. Data variation (time effect) for the in-water variables during three consecutive stroke cycles (i.e., v_mean_, v_peak_, dv, UST, F_mean_, F_peak_, dF) was assessed with repeated measures ANOVA, and the Bonferroni post-hoc test was used to verify hypothetical significant differences between each pairwise (p < 0.05). The effect size index (eta square—η^2^) was computed and interpreted as: (1) without effect if 0 < η^2^ ≤ 0.04; (2) minimum if 0.04 < η^2^ ≤ 0.25; (3) moderate if 0.25 < η^2^ ≤ 0.64 and; (4) strong if η^2^ > 0.64.

The swim speed (v_mean_) achieved by each upper-limb was defined as the dependent variable. Remaining variables of both upper-limbs (arm, forearm, HSA, handgrip, v_peak_, dv, UST, F_mean_, F_peak_, and dF) were defined as independent or predictor variables. The analysis was performed using the multilevel modelling software MLwiN. Multilevel modelling is an extension of ordinary multiple regression where the data have a hierarchical or clustered structure. The hierarchy consists of units or measurements grouped at different levels. In the current study, the swimmers are assumed to be a random sample, represent the level 2 units, with the swimmers’ repeated measurements (three consecutive stroke cycles), being the level 1 units. A multicollinearity phenomenon was not detected since the independent variables were all computed independently from the dependent one.
